# Non-parametric and semi-parametric support estimation using SEquential RESampling random walks on biomolecular sequences

**DOI:** 10.1186/s13015-020-00167-0

**Published:** 2020-04-16

**Authors:** Wei Wang, Jack Smith, Hussein A. Hejase, Kevin J. Liu

**Affiliations:** 1 Department of Computer Science and Engineering, Michigan State University, East Lansing, MI 48824 USA; 2 Simons Center for Quantitative Biology, Cold Spring Harbor Laboratory, Cold Spring Harbor, NY 11724 USA

**Keywords:** Statistical support, Non-parametric, Semi-parametric, Resampling, Bootstrap, Multiple sequence alignment, Random walk

## Abstract

Non-parametric and semi-parametric resampling procedures are widely used to perform support estimation in computational biology and bioinformatics. Among the most widely used methods in this class is the standard bootstrap method, which consists of random sampling with replacement. While not requiring assumptions about any particular parametric model for resampling purposes, the bootstrap and related techniques assume that sites are independent and identically distributed (i.i.d.). The i.i.d. assumption can be an over-simplification for many problems in computational biology and bioinformatics. In particular, sequential dependence within biomolecular sequences is often an essential biological feature due to biochemical function, evolutionary processes such as recombination, and other factors. To relax the simplifying i.i.d. assumption, we propose a new non-parametric/semi-parametric sequential resampling technique that generalizes “Heads-or-Tails” mirrored inputs, a simple but clever technique due to Landan and Graur. The generalized procedure takes the form of random walks along either aligned or unaligned biomolecular sequences. We refer to our new method as the SERES (or “SEquential RESampling”) method. To demonstrate the performance of the new technique, we apply SERES to estimate support for the multiple sequence alignment problem. Using simulated and empirical data, we show that SERES-based support estimation yields comparable or typically better performance compared to state-of-the-art methods.

## Introduction

Resampling methods are widely used throughout computational biology and bioinformatics as a means for assessing statistical support. At a high level, resampling-based support estimation procedures consist of a methodological pipeline: resampled replicates are generated, inference/analysis is performed on each replicate, and results are then compared across replicates. Among the most widely used resampling methods are non-parametric approaches including the standard bootstrap method [6], which consists of random sampling with replacement. We will refer to the standard bootstrap method as the bootstrap method for brevity. Unlike parametric methods, non-parametric approaches need not assume that a particular parametric model is applicable to a problem at hand. However, the bootstrap and other widely used non-parametric approaches assume that observations are independent and identically distributed (i.i.d.).

In the context of biomolecular sequence analysis, there are a variety of biological factors that conflict with this assumption. These include evolutionary processes that cause intra-sequence dependence (e.g., recombination) and functional dependence among biomolecular sequence elements and motifs. Felsenstein presciently noted these limitations when he proposed the application of the bootstrap to phylogenetic inference: “A more serious difficulty is lack of independence of the evolutionary processes in different characters. $\ldots$ For the purposes of this paper, we will ignore these correlations and assume that they cause no problems; in practice, they pose the most serious challenge to the use of bootstrap methods.” (reproduced from p. 785 of [7]).

To relax the simplifying assumption of i.i.d. observations, Landan and Graur [11] introduced the Heads-or-Tails (HoT) technique for the specific problem of multiple sequence alignment (MSA) support estimation. The idea behind HoT is simple but quite powerful: inference/analysis should be repeatable whether an MSA is read either from left-to-right or from right-to-left—i.e., in either heads or tails direction, respectively. While HoT resampling preserves intra-sequence dependence, it is limited to two replicates, which is far fewer than typically needed for reasonable support estimation; often, hundreds of resampled replicates or more are used in practice. Subsequently developed support estimation procedures increased the number of possible replicates by augmenting HoT with bootstrapping, parametric resampling, and domain-specific techniques (e.g., progressive MSA estimation) [12, 18, 20]. The combined procedures were shown to yield comparable or improved support estimates relative to the original HoT procedure [20] as well as other state-of-the-art parametric and domain-specific methods [10, 16], at the cost of some of the generalizability inherent to non-parametric approaches. In this study, we revisit the central question that HoT partially addressed: how can we resample many non-parametric replicates that account for dependence within a sequence of observations, and how can such techniques be used to derive improved support estimates for biomolecular sequence analysis?

## Methods

In our view, a more general statement of HoT’s main insight is the following, which we refer to as the “neighbor preservation property”: a neighboring observation is still a neighbor, whether reading an observation sequence from the left or the right. In other words, the key property needed for non-parametric resampling is preservation of neighboring bases within the original sequences, where any pair of bases that appear as neighbors in a resampled sequence must also be neighbors in the corresponding original sequence. To obtain many resampled replicates that account for intra-sequence dependence while retaining the neighbor preservation property, we propose a random walk procedure which generalizes a combination of the bootstrap method and the HoT method. We refer to the new resampling procedure as SERES (“SEquential RESampling”). Note that the neighbor preservation property is necessary but not sufficient for statistical support estimation. Other important properties include computational efficiency of the resampling procedure and unbiased sampling of observations within the original observation sequence.

SERES walks can be performed on both aligned and unaligned sequence inputs. We discuss the case of aligned inputs first, since it is simpler than the case of unaligned inputs.

### SERES walks on aligned sequences

Detailed pseudocode for a non-parametric SERES walk on a fixed MSA is shown in Additional file 1: Additional methods section: Algorithm 1.

The random walk is performed on the sequence of aligned characters (i.e., MSA sites). The starting point for the walk is chosen uniformly at random from the alignment sites, and the starting direction is also chosen uniformly at random. The random walk then proceeds in the chosen direction with non-deterministic reversals, or direction changes, that occur with probability $\gamma$; furthermore, reversals occur with certainty at the start and end of the fixed MSA. Aligned characters are sampled during each step of the walk. The random walk ends once the number of sampled characters is equal to the fixed MSA length.

The long-term behavior of an infinitely long SERES random walk can be described by a second-order Markov chain. Certain special cases (e.g., $\gamma =0.5$) can be described using a first-order Markov chain.

In theory, a finite-length SERES random walk can exhibit biased sampling of sites since reversal occurs with certainty at the start and end of the observation sequence, whereas reversal occurs with probability $\gamma$ elsewhere. However, for practical choices of walk length and reversal probability $\gamma$, sampling bias is expected to be minimal.

### SERES walks on unaligned sequences

**Fig. 1:**
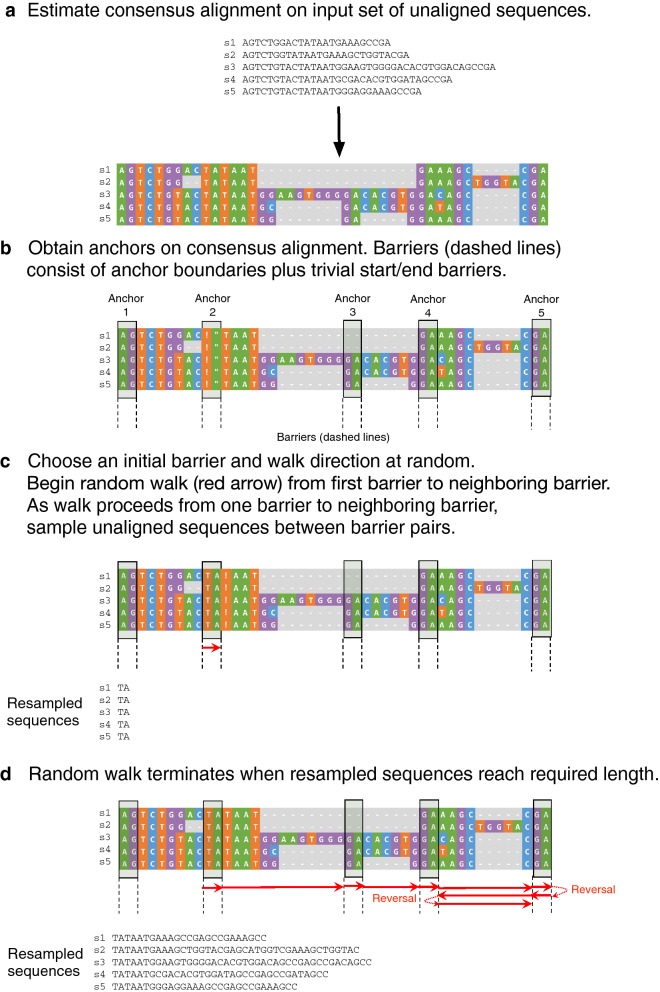
Illustrated example of SERES resampling random walk on unaligned sequences. Detailed pseudocode is provided in Additional file 1: Additional methods section: Algorithm 2. **a** The resampling procedure begins with the estimation of a consensus alignment on the input set of unaligned sequences. **b** A set of conservative anchors is then obtained using the consensus alignment, and anchor boundaries define a set of barriers (including two trivial barriers—one at the start of the sequences and one at the end of the sequences). **c** The SERES random walk is conducted on the set of barriers. The walk begins at a random barrier and proceeds in a random direction to the neighboring barrier. The walk reverses with certainty when the trivial start/end barriers are encountered; furthermore, the walk direction can randomly reverse with probability $\gamma$. As the walk proceeds from barrier to barrier, unaligned sequences are sampled between neighboring barrier pairs. **d** The resampling procedure terminates when the resampled sequences meet a specified sequence length threshold

Detailed pseudocode for SERES resampling of unaligned sequences is shown in Additional file 1: Additional methods section: Algorithm 2. Figure 1 provides an illustrated example.

The procedure begins with estimating a set of anchors—sequence regions that exhibit high sequence similarity—which enable resampling synchronization across unaligned sequences. A conservative approach for identifying anchors would be to use highly similar regions that appear in the strict consensus of multiple MSA estimation methods. In practice, we found that highly similar regions within a single guide MSA produced reasonable anchors. We used the average normalized Hamming distance (ANHD) as our similarity measure, where indels are treated as mismatches.

Unaligned sequence indices corresponding to the start and end of each anchor serve as “barriers” in much the same sense as in parallel computing: asynchronous sequence reads occur between barrier pairs along a current direction (left or right), and a random walk is conducted on barrier space in a manner similar to a SERES walk on a sequence of aligned characters. The set of barriers also includes trivial barriers at the start and end of the unaligned sequences. The random walk concludes once the unaligned sequences in the resampled replicate have sufficient length; our criterion requires that the longest resampled sequence has minimum length that is a multiple maxReplicateLengthFactor of the longest input sequence length.

Technically, the anchors in our study make use of parametric MSA estimation and the rest of the SERES walk is non-parametric. The overall procedure is therefore semi-parametric (although see “Conclusions” for an alternative).

### Performance study

Our study evaluated the performance of SERES-based support estimation in the context of MSA support estimation. Of course, there are many other applications for non-parametric/semi-parametric support estimation—too many to investigate in one study. We focus on this application since the multiple sequence alignment problem is considered to be a classical problem in computational biology and bioinformatics and MSAs are used as inputs for a variety of important computational problems throughout computational biology and bioinformatics (e.g., phylogenetics and phylogenomics, proteomics, comparative genomics, etc.). It is well known that MSA quality has a major impact on downstream analysis [11, 14, 15]. We also note that the need to quantify support in the context of MSA estimation bears upon the critical issues of scientific rigor and reproducibility.

#### Computational methods

We examined the problem of evaluating support in the context of MSA estimation. The problem input consists of an estimated MSA *A* which has a corresponding set of unaligned sequences *S*. The problem output consists of support estimates for each nucleotide-nucleotide homology in *A*, where each support estimate is on the unit interval. Note that this computational problem is distinct from the full MSA estimation problem.

There are a variety of existing methods for MSA support estimation. The creators of HoT and their collaborators subsequently developed alignment-specific parametric resampling techniques [12] and then combined the two to obtain two new semi-parametric approaches: GUIDANCE [18] (which we will refer to as GUIDANCE1) and GUIDANCE2 [20]. Other parametric MSA support estimation methods include PSAR [10] and T-Coffee [16].

We focus on GUIDANCE1 and GUIDANCE2, which subsume HoT and have been demonstrated to have comparable or better performance relative to other state-of-the-art methods [20]. We used MAFFT for re-estimation on resampled replicates, since it has been shown to be among the most accurate progressive MSA methods to date [9, 15].

We then used SERES to perform resampling in place of the standard bootstrap that is used in the first step of GUIDANCE1/GUIDANCE2. Re-estimation was performed on 100 SERES replicates—each consisting of a set of unaligned sequences—using MAFFT with default settings, which corresponds to the FFT-NS-2 algorithm for progressive alignment. The SERES resampling procedure used a reversal probability $\gamma =0.5$, which is equivalent to selecting a direction uniformly at random (UAR) at each step of the random walk; each SERES replicate utilized a total of $\lfloor \frac{k}{20} \rfloor$ anchors with anchor size of 5 bp and a minimum distance between neighboring anchors of 25 bp, where *k* is the length of the input alignment *A*. All downstream steps of GUIDANCE1/GUIDANCE2 were then performed using the re-estimated alignments as input.

To further explore the impact of our algorithmic design choices, we included additional experiments which varied the parameter settings used to perform SERES-based support estimation. Each set of experiments manipulated one parameter setting—either the number of anchors, anchor length, or the method used for estimating the input MSA—but otherwise used default settings for SERES-based support estimation. The number of anchors was selected from the set $\{3, 5, 20, 50, 100\}$. Anchor length in bp was chosen from the set $\{3, 5, 10, 30, 50\}$. Three different methods were used for estimating an input MSA: ClustalW [13], MAFFT [9], and FSA [2].

#### Simulated datasets

Model trees and sequences were simulated using INDELible [8]. First, non-ultrametric model trees with either 10 or 50 taxa were sampled using the following procedure. Model trees were generated under a birth-death process [21], branch lengths were chosen UAR from the interval (0, 1), and the model tree height was re-scaled from its original height $h_0$ to a desired height *h* by multiplying all branch lengths by the factor $h/h_0$. Next, sequences were evolved down each model tree under the General Time-Reversible (GTR) model of substitution [19] and the indel model of Fletcher and Yang [8], where the root sequence had length of 1 kb. We used the substitution rates and base frequencies from the study of Liu et al. [15], which were based upon empirical analysis of the nematode Tree of Life. Sequence insertions/deletions occurred at rate $r_i$, and we used the medium gap length distribution from the study of Liu et al. [15].

The model parameter values used for simulation and summary statistics computed on the simulated datasets are shown in Table 1. Each combination of model parameter values constitutes a model condition. Model conditions are enumerated in order of generally increasing sequence divergence, as reflected by ANHD. For each model condition, the simulation procedure was repeated to generate twenty replicate datasets.

**Table 1:** Medium-gap-length model conditions: parameter values and summary statistics

Model condition	Number of taxa	Tree height	Insertion/deletion probability	NHD	Gappiness	True align length
10.A	10	0.4	0.13	0.297	0.474	1965.3
10.B	10	0.7	0.1	0.394	0.512	2165.1
10.C	10	1	0.06	0.514	0.526	2162.8
10.D	10	1.6	0.031	0.599	0.485	1874.4
10.E	10	4.3	0.013	0.693	0.465	1849.3
50.A	50	0.45	0.06	0.281	0.516	2043.5
50.B	50	0.7	0.03	0.398	0.475	1935.5
50.C	50	1	0.02	0.514	0.498	2047.6
50.D	50	1.8	0.012	0.594	0.471	1945.0
50.E	50	4.3	0.004	0.688	0.459	1890.2

The main simulations in our study utilized the medium gap length distribution from the study of Liu et al. [15]. The model condition parameters consist of the number of taxa, model tree height, and insertion/deletion probability. Each model condition corresponds to a distinct set of model parameter values. The 10-taxon model conditions are named 10.A through 10.E in order of generally increasing sequence divergence; the 50-taxon model conditions are named 50.A through 50.E similarly. The following table columns list average summary statistics for each model condition ($n=20$). “NHD” is the average normalized Hamming distance of a pair of aligned sequences in the true alignment. “Gappiness” is the percentage of true alignment cells which consists of indels. “True align length” is the length of the true alignment

To explore the impact of gap length distribution, our study also included 10-taxon model conditions which utilized the long gap length distribution from the study of Liu et al. [15] in place of the medium gap length distribution that was used elsewhere in our simulation study. Parameter values and summary statistics for the long-gap-length model conditions are shown in Table 2.

**Table 2:** Long-gap-length model conditions: parameter values and summary statistics

Model condition	Tree height	Insertion/deletion probability	NHD	Gappiness	True align length	Est align length	SP-FN	SP-FP
10.long.A	0.4	0.13	0.276	0.440	1804.8	1433.7	0.272	0.315
10.long.B	0.7	0.1	0.363	0.481	1926.7	1447.8	0.381	0.426
10.long.C	1	0.06	0.455	0.456	1853.5	1413.3	0.510	0.537
10.long.D	1.6	0.031	0.542	0.432	1754.1	1403.1	0.725	0.729
10.long.E	4.3	0.013	0.660	0.445	1811.0	1560.1	0.899	0.897

Our simulation study included additional 10-taxon model conditions that utilized the long gap length distribution from the study of Liu et al. [15]. The model parameters consisted of model tree height and insertion/deletion probability, and each model condition corresponds to a distinct set of model parameter values. The long-gap-length model conditions are named 10.long.A through 10.long.E in order of generally increasing sequence divergence. The following table columns list average summary statistics for each model condition ($n=20$). “NHD” is the average normalized Hamming distance of a pair of aligned sequences in the true alignment. “Gappiness” is the percentage of true alignment cells which consists of indels. “True align length” is the length of the true alignment. “Est align length” is the length of the MAFFT-estimated alignment [9] which was provided as input to the support estimation methods. “SP-FN” and “SP-FP” are the proportion of homologies that appear in the true alignment but not in the MAFFT-estimated alignment and vice versa, respectively

The MSA support estimation problem under study requires an MSA as input. Summary statistics for the estimated alignments used as input are provided in Table 3.

**Table 3:** Medium-gap-length model conditions: estimated alignment statistics

Model condition	Est align length	SP-FN	SP-FP
***MAFFT***			
10.A	1552.3	0.294	0.341
10.B	1563.5	0.483	0.533
10.C	1554.0	0.657	0.684
10.D	1507.5	0.747	0.752
10.E	1612.8	0.945	0.943
50.A	1785.7	0.086	0.088
50.B	1714.2	0.105	0.102
50.C	1703.1	0.245	0.230
50.D	1712.2	0.455	0.419
50.E	2319.2	0.963	0.948

Model condition	Est align length	SP-FN	SP-FP
***ClustalW***			
10.A	1208.5	0.497	0.556
10.B	1186.2	0.624	0.684
10.C	1144.8	0.711	0.754
10.D	1105.7	0.756	0.786
10.E	1060.1	0.896	0.906

Model condition	Est align length	SP-FN	SP-FP
***FSA***			
10.A	2289.3	0.334	0.124
10.B	3418.5	0.585	0.164
10.C	4506.6	0.729	0.211
10.D	5000.9	0.800	0.223
10.E	6657.1	0.907	0.531

The MSA support estimation problem requires an input MSA. MAFFT [9] was used to estimate an input MSA for all model conditions in our study. Our study also included ClustalW [13] and FSA [2] alignments to explore the impact of input alignment quality on downstream support estimation. The following table columns list average statistics for estimated alignments on each model condition ($n=20$). “Est align length” is the estimated alignment length. “SP-FN” and “SP-FP” are the proportion of homologies that appear in the true alignment but not in the estimated alignment and vice versa, respectively

The performance of the MSA support estimation methods in our study was evaluated using receiver operating characteristic (ROC) curves, precision-recall (PR) curves, and area under ROC and PR curves (ROC-AUC and PR-AUC, respectively). Consistent with other studies of MSA support estimation techniques [18, 20], the MSA support estimation problem in our study entails annotation of nucleotide-nucleotide homologies in the estimated alignment; thus, homologies that appear in the true alignment but not the estimated alignment are not considered. For this reason, the confusion matrix quantities used for ROC and PR calculations are defined as follows. True positives (TP) are the set of nucleotide-nucleotide homologies that appear in the true alignment and the estimated alignment with support value greater than or equal to a given threshold, false positives (FP) are the set of nucleotide-nucleotide homologies that appear in the estimated alignment with support value greater than or equal to a given threshold but do not appear in the true alignment, false negatives (FN) are the set of nucleotide-nucleotide homologies that appear in the true alignment but appear in the estimated alignment with support value below a given threshold, and true negatives (TN) are the set of nucleotide-nucleotide homologies that do not appear in the true alignment and appear in the estimated alignment with support value below a given threshold. The ROC curve plots the true positive rate ($|\text {TP}| / (|\text {TP}| + |\text {FN}|)$) versus the false positive rate ($|\text {FP}| / (|\text {FP}| + |\text {TN}|)$). The PR curve plots the true positive rate versus precision ($|\text {TP}| / (|\text {TP}| + |\text {FP}|)$). Varying the support threshold yields different points along these curves. Custom scripts were used to perform confusion matrix calculations. ROC curve, PR curve, and AUC calculations were performed using the scikit-learn Python library [17].

#### Empirical datasets

We downloaded empirical benchmarks from the Comparative RNA Web (CRW) Site database, which can be found at http://www.rna.icmb.utexas.edu [3]. In brief, the CRW database includes ribosomal RNA sequence datasets than span a range of dataset sizes and evolutionary divergence. We focused on datasets where high-quality reference alignments are available; the reference alignments were produced using intensive manual curation and analysis of heterogeneous data, including secondary structure information. We selected primary 16S rRNA, primary 23S rRNA, primary intron, and seed alignments with at most 250 sequences. Aligned sequences with 99% or more missing data and/or indels were omitted from analysis. Summary statistics for the empirical benchmarks are shown in Table 4.

**Table 4:** Empirical dataset summary statistics

Dataset	Number of taxa	NHD	Gappiness	Ref align length	Est align length	SP-FN	SP-FP
IGIA	110	0.606	0.915	10,368	6675	0.734	0.784
IGIB	202	0.579	0.910	10,633	7379	0.825	0.864
IGIC2	32	0.533	0.700	4243	3514	0.689	0.715
IGID	21	0.719	0.782	5061	3023	0.874	0.904
IGIE	249	0.451	0.838	2751	2775	0.393	0.376
IGIIA	174	0.668	0.814	6406	7005	0.816	0.800
PA23	142	0.293	0.267	3991	3552	0.078	0.077
PE23	117	0.300	0.612	9436	10,083	0.202	0.213
PM23	102	0.361	0.797	10,999	8803	0.262	0.288
SA16	132	0.212	0.205	1866	1673	0.031	0.028
SA23	144	0.304	0.460	4048	3678	0.077	0.081

The empirical study made use of reference alignments (“Ref align”) from the CRW database [3]. The reference alignments were curated using heterogeneous data including secondary structure information. The column description is identical to Table 2, where the empirical study made use of reference alignments in lieu of the simulation study’s true alignments

### Computational resources used and software/data availability

All computational analyses were run on computing facilities in Michigan State University’s High Performance Computing Center. We used compute nodes in the intel16-k80 cluster, each of which had a 2.4 GHz 14-core Intel Xeon E5-2680v4 processor with 256 GiB of main memory. Open-source software and open data can be found at https://gitlab.msu.edu/liulab/SERES-Scripts-Data.

## Results

### Simulation study

On the medium-gap-length model conditions, SERES-based resampling and re-estimation yielded improved MSA support estimates compared to GUIDANCE1 and GUIDANCE2, two state-of-the-art methods, where performance was measured by PR-AUC or ROC-AUC (Table 5). In all cases, PR-AUC or ROC-AUC improvements were statistically significant (corrected pairwise t-test or DeLong et al. [5] test, respectively; $n=20$ and $\alpha =0.05$). The observed performance improvement was robust to several experimental factors: dataset size, increasing sequence divergence due to increasing numbers of substitutions, insertions, and deletions, and the choice of alignment-specific parametric support estimation techniques (i.e., the parametric approaches used by either GUIDANCE1 or GUIDANCE2) that were used in combination with SERES-based support estimation.

**Table 5:** Support estimation method performance on main model conditions

Model condition	PR-AUC (%)		Pairwise t-test corrected q-value	ROC-AUC (%)		DeLong et al. test corrected q-value
	GUIDANCE1	SERES + GUIDANCE1		GUIDANCE1	SERES + GUIDANCE1	
10.A	88.74	*91.17*	$5.4 \times 10^{-7}$	80.22	*85.57*	$<10^{-10}$
10.B	82.21	*86.26*	$1.5 \times 10^{-6}$	84.83	*88.66*	$<10^{-10}$
10.C	76.23	*83.49*	$1.9 \times 10^{-4}$	86.98	*91.23*	$<10^{-10}$
10.D	74.65	*85.81*	$1.9 \times 10^{-4}$	88.55	*93.72*	$<10^{-10}$
10.E	42.61	*59.20*	$3.1 \times 10^{-4}$	82.24	*87.40*	$<10^{-10}$
50.A	98.22	*98.92*	$5.3 \times 10^{-10}$	83.09	*90.64*	$<10^{-10}$
50.B	97.84	*98.69*	$2.8 \times 10^{-9}$	82.85	*90.39*	$<10^{-10}$
50.C	95.08	*96.80*	$5.6 \times 10^{-8}$	85.54	*90.64*	$<10^{-10}$
50.D	90.79	*95.75*	$5.3 \times 10^{-6}$	88.89	*94.56*	$<10^{-10}$
50.E	62.47	*79.14*	$8.0 \times 10^{-10}$	91.02	*93.23*	$<10^{-10}$

Model condition	PR-AUC (%)		Pairwise t-test corrected q-value	ROC-AUC (%)		DeLong et al. test corrected q-value
	GUIDANCE2	SERES + GUIDANCE2		GUIDANCE2	SERES + GUIDANCE2	
10.A	92.55	*93.33*	$7.4 \times 10^{-6}$	87.17	*88.34*	$<10^{-10}$
10.B	88.08	*89.31*	$8.4 \times 10^{-4}$	89.45	*90.56*	$<10^{-10}$
10.C	84.28	*86.86*	$3.1 \times 10^{-4}$	91.36	*92.88*	$<10^{-10}$
10.D	86.03	*88.75*	$1.9 \times 10^{-4}$	93.34	*94.69*	$<10^{-10}$
10.E	51.17	*62.30*	$1.3 \times 10^{-3}$	86.00	*88.28*	$<10^{-10}$
50.A	98.98	*99.14*	$5.3 \times 10^{-6}$	91.17	*92.50*	$<10^{-10}$
50.B	98.79	*98.96*	$1.5 \times 10^{-6}$	91.24	*92.44*	$<10^{-10}$
50.C	96.86	*97.45*	$3.2 \times 10^{-7}$	90.81	*92.31*	$<10^{-10}$
50.D	94.04	*96.23*	$1.5 \times 10^{-5}$	92.67	*95.09*	$<10^{-10}$
50.E	72.61	*81.47*	$1.5 \times 10^{-8}$	92.94	*94.22*	$<10^{-10}$

Results are shown for five 10-taxon model conditions (named 10.A through 10.E in order of generally increasing sequence divergence) and five 50-taxon model conditions (similarly named 50.A through 50.E). We evaluated the performance of two state-of-the-art methods for MSA support estimation—GUIDANCE1 [18] and GUIDANCE2 [20]—versus re-estimation on SERES and parametrically resampled replicates (using parametric techniques from either GUIDANCE1 or GUIDANCE2) (see “Methods” section for details.) We calculated each method’s precision-recall (PR) and receiver operating characteristic (ROC) curves. Performance is evaluated based upon aggregate area under curve (AUC) across all replicates for a model condition ($n=20$). The top rows show AUC comparisons of GUIDANCE1 (“GUIDANCE1”) vs. SERES combined with parametric techniques from GUIDANCE1 (“SERES + GUIDANCE1”), and the bottom rows show AUC comparisons of GUIDANCE2 (“GUIDANCE2”) vs. SERES combined with parametric techniques from GUIDANCE2 (“SERES + GUIDANCE2”); for each model condition and pairwise comparison, the best AUC is shown in italics. Statistical significance of PR-AUC or ROC-AUC differences was assessed using a one-tailed pairwise t-test or DeLong et al. [5] test, respectively, and multiple test correction was performed using the method of Benjamini and Hochberg [1]. Corrected q-values are reported ($n=20$) and all were significant ($\alpha =0.05$)

Compared to dataset size, sequence divergence had a relatively greater quantitative impact on each method’s performance. For each dataset size (10 or 50 taxa), PR-AUC differed by at most 3% on the least divergent model condition. The SERES-based method’s performance advantage grew as sequence divergence increased—to as much as 28%—and the largest performance advantages were seen on the most divergent datasets in our study. The most divergent datasets were also the most challenging. For each method, PR-AUC generally degraded as sequence divergence increased; however, the SERES-based method’s PR-AUC degraded more slowly compared to the non-SERES-based method. Consistent with the study of Sela et al. [20], GUIDANCE2 consistently outperformed GUIDANCE1 on each model conditions and using either AUC measure. The performance improvement of SERES + GUIDANCE1 over GUIDANCE1 was generally greater than that seen when comparing SERES + GUIDANCE2 and GUIDANCE2; furthermore, the PR-AUC-based corrected q-values were more significant for the former compared to the latter in all cases except for the 10.D model condition, where the corrected q-values were comparable. Finally, while the SERES-based method consistently yielded performance improvements over the corresponding non-SERES-based method regardless of the choice of performance measure (either PR-AUC or ROC-AUC), the PR-AUC difference was generally larger than the ROC-AUC difference, especially on more divergent model conditions.

In terms of average runtime on the 10-taxon and 50-taxon model conditions, SERES + GUIDANCE2 added overhead of at most 1.4 min and 6.5 min relative to GUIDANCE2, respectively (Additional file 1: Figure S1). The average runtime overhead of SERES + GUIDANCE1 relative to GUIDANCE1 was at most 1 min and 5 min on the 10-taxon and 50-taxon model conditions, respectively. In terms of average memory usage on 10-taxon and 50-taxon model conditions, SERES + GUIDANCE2 adds at most 0.034 GiB and 0.871 GiB overhead relative to GUIDANCE2, respectively (Additional file 1: Figure S2). A similar outcome was observed when comparing SERES + GUIDANCE1 and GUIDANCE1. On average, all methods in the simulation study completed analysis of each replicate dataset in less than half an hour and with less than 1 GiB of main memory usage.

Performance comparisons on the long-gap-length model conditions (Table 6) were largely similar to the medium-gap-length model conditions. SERES + GUIDANCE2 consistently returned significant improvements in PR-AUC and ROC-AUC relative to GUIDANCE2 (corrected pairwise t-test or DeLong et al. [5] test, respectively; $n=20$ and $\alpha =0.05$). Furthermore, SERES + GUIDANCE2’s PR-AUC advantage relative to GUIDANCE2 tended to improve on more divergent model conditions. With a single exception, PR-AUC improvement of SERES + GUIDANCE2 over GUIDANCE2 was similar (within a single percentage point) when comparing medium-gap-length/long-gap-length model condition pairs that were otherwise equivalent (e.g., 10.A and 10.long.A); a similar finding was observed for ROC-AUC measurements. The single exception occurred on the 10.D and 10.long.D model conditions, where a larger PR-AUC performance improvement by the SERES-based method was seen on the 10.long.D model condition versus the 10.D model condition.

**Table 6:** Support estimation method performance on long-gap-length model conditions

Model condition	PR-AUC (%)		
	GUIDANCE2	SERES + GUIDANCE2	Pairwise t-test corrected q-value
10.long.A	92.32	*92.94*	$9.7 \times 10^{-4}$
10.long.B	90.62	*91.64*	$3.3 \times 10^{-6}$
10.long.C	85.10	*87.93*	$9.7 \times 10^{-4}$
10.long.D	79.22	*86.18*	$9.7 \times 10^{-4}$
10.long.E	67.63	*78.48*	$9.7 \times 10^{-4}$

Model condition	ROC-AUC (%)		
	GUIDANCE2	SERES + GUIDANCE2	DeLong et al. test corrected q-value
10.long.A	89.99	*90.99*	$<10^{-10}$
10.long.B	91.84	*93.02*	$<10^{-10}$
10.long.C	93.14	*94.59*	$<10^{-10}$
10.long.D	93.89	*96.13*	$<10^{-10}$
10.long.E	92.62	*94.38*	$<10^{-10}$

The performance of GUIDANCE2 and SERES + GUIDANCE2 is compared across model conditions 10.long.A through 10.long.E (named in order of generally increasing sequence divergence). Aggregate PR-AUC and ROC-AUC are reported across all replicate datasets in a model condition ($n=20$), and the best AUC for each pairwise method comparison on a model condition is shown in italics. Statistical significance of PR-AUC or ROC-AUC differences was assessed using a one-tailed pairwise t-test or DeLong et al. [5] test, respectively, and multiple test correction was performed using the method of Benjamini and Hochberg [1]. Corrected q-values are reported ($n=20$) and all were significant ($\alpha =0.05$)

**Fig. 2:**
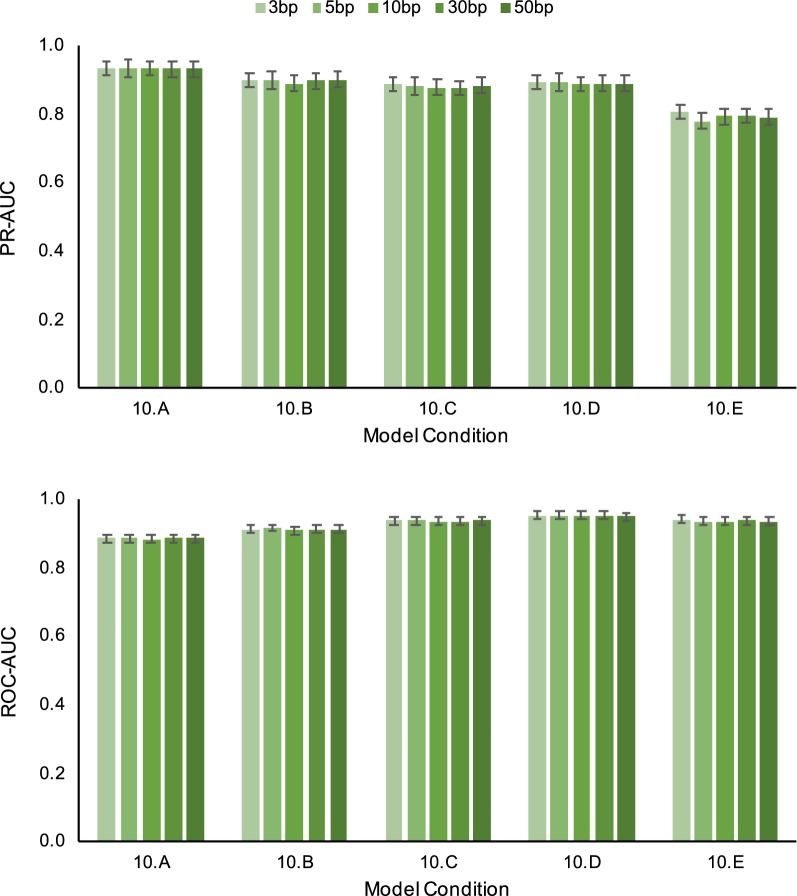
SERES + GUIDANCE2 performance using different choices for anchor length. Results are shown for five 10-taxon medium-gap-length model conditions (named 10.A through 10.E in order of generally increasing sequence divergence). We evaluated the performance of SERES + GUIDANCE2 where anchor length in bp was either 3, 5, 10, 30, or 50. We calculated each method’s precision-recall (PR) and receiver operating characteristic (ROC) curves. Performance is evaluated based upon aggregate area under curve (AUC) across all replicates for a model condition ($n=20$)

**Fig. 3:**
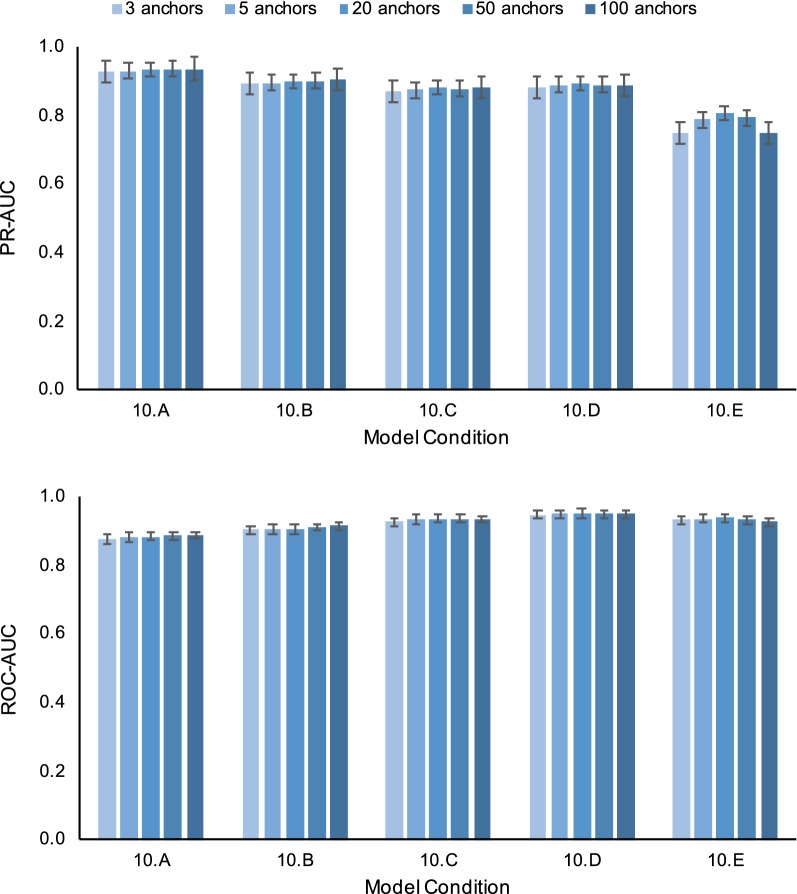
SERES + GUIDANCE2 performance using different choices for the number of anchors. We evaluated the performance of SERES + GUIDANCE2 where the number of anchors used was either 3, 5, 20, 50, or 100. Otherwise, figure layout and description are identical to Fig. 2

We also conducted additional experiments to study the impact of three algorithmic design choices. Table 7 shows performance results for SERES + GUIDANCE2 using alternative methods for estimating an input MSA. Note that the three MSA methods in our study returned varying input alignment quality; relative to the other two MSA methods, FSA returned lower average SP-FP and was best or close to best in terms of average SP-FN (Table 3). Downstream support estimation PR-AUC tended to reflect input alignment quality. While PR-AUC tended to degrade as model conditions became more divergent, smaller PR-AUC reductions were seen when using FSA as input alignment versus MAFFT or ClustalW. SERES + GUIDANCE2’s PR-AUC and ROC-AUC performance advantage over GUIDANCE2 was robust to input alignment quality: it returned PR-AUC and ROC-AUC improvements when annotating more accurate input alignments (i.e., FSA alignments) as well as less accurate input alignments (i.e., the MAFFT and ClustalW alignments). Results for algorithmic design experiments using differing choices for anchor length and numbers of anchors are shown in Figs. 2 and 3, respectively. SERES + GUIDANCE2 returned comparable PR-AUC and ROC-AUC regardless of anchor length used for SERES resampling. The average ROC-AUC difference for different choices of anchor length was less than 0.01 for all model conditions. The largest PR-AUC difference was 0.058 on the 10.E model condition; in comparison, SERES + GUIDANCE2’s PR-AUC improvement over GUIDANCE2 was 0.28 on the 10.E model condition. A similar outcome was seen on experiments involving different choices for the number of anchors, with one exception: on the most divergent 10.E model condition, an intermediate number of anchors (about 20) yielded the best PR-AUC.

**Table 7:** SERES + GUIDANCE2 performance using alternative methods for estimating an input MSA

Model condition	PR-AUC (%)					
	ClustalW			FSA		
	GUIDANCE2	SERES + GUIDANCE2	Pairwise t-test corrected q-value	GUIDANCE2	SERES + GUIDANCE2	Pairwise t-test corrected q-value
10.A	95.37	*95.78*	$2.8 \times 10^{-3}$	96.36	*96.55*	$8.6 \times 10^{-3}$
10.B	92.30	*92.95*	$8.2 \times 10^{-4}$	95.40	*95.87*	$4.9 \times 10^{-3}$
10.C	89.36	*91.23*	$1.7 \times 10^{-4}$	95.32	*96.06*	$2.7 \times 10^{-3}$
10.D	88.53	*90.45*	$8.8 \times 10^{-5}$	96.21	*96.87*	$2.1 \times 10^{-3}$
10.E	73.96	*76.50*	$8.2 \times 10^{-4}$	90.23	*92.51*	$8.6 \times 10^{-3}$

Model condition	ROC-AUC (%)					
	ClustalW			FSA		
	GUIDANCE2	SERES + GUIDANCE2	DeLong et al. test corrected q-value	GUIDANCE2	SERES + GUIDANCE2	DeLong et al. test corrected q-value
10.A	96.99	*97.23*	$<10^{-10}$	80.85	*81.61*	$<10^{-10}$
10.B	96.64	*96.94*	$<10^{-10}$	81.31	*82.89*	$<10^{-10}$
10.C	96.27	*96.88*	$<10^{-10}$	84.48	*86.56*	$<10^{-10}$
10.D	95.78	*96.65*	$<10^{-10}$	88.63	*90.37*	$<10^{-10}$
10.E	89.84	*90.80*	$<10^{-10}$	89.10	*90.83*	$<10^{-10}$

Input MSAs in these experiments were estimated using either ClustalW [13] or FSA [2] (MAFFT was used to estimate input MSAs throughout the rest of our study.) Results are shown for model conditions 10.A through 10.E (named in order of generally increasing sequence divergence). The best AUC for each pairwise method comparison on a model condition is shown in italics. Otherwise, table layout and description are identical to Table 6

### Empirical study

Relative to GUIDANCE1 or GUIDANCE2, SERES-based support estimates consistently returned higher AUC on all datasets—primary, seed, and intronic—with a single exception: the comparison of SERES + GUIDANCE2 and GUIDANCE2 on the intronic IGIC2 dataset, where the PR-AUC and ROC-AUC differences were 1.17% and 2.12%, respectively (Table 8). For each pairwise comparison of methods (i.e., SERES + GUIDANCE1 vs. GUIDANCE1 or SERES + GUIDANCE2 vs. GUIDANCE2), the SERES-based method returned relatively larger PR-AUC improvements on datasets with greater sequence divergence, as measured by ANHD and gappiness. In particular, PR-AUC improvements were less than 1% on seed and primary non-intronic datasets. Intronic datasets yielded PR-AUC improvements of as much as 13.87%. Observed AUC improvements of SERES + GUIDANCE1 over GUIDANCE1 were relatively greater than those seen for SERES + GUIDANCE2 in comparison to GUIDANCE2. Finally, GUIDANCE2 consistently returned higher AUC relative to GUIDANCE1, regardless of whether PR or ROC curves were the basis for AUC comparison.

**Table 8:** Empirical study results

Dataset	PR-AUC (%)		ROC-AUC (%)	
	GUIDANCE1	SERES + GUIDANCE1	GUIDANCE1	SERES + GUIDANCE1
IGIA	62.67	*69.28*	89.50	*91.62*
IGIB	73.60	*87.47*	94.49	*97.39*
IGIC2	72.67	*75.36*	82.25	*83.87*
IGID	63.74	*76.30*	95.10	*96.73*
IGIE	93.56	*95.42*	90.08	*93.30*
IGIIA	73.03	*83.06*	86.49	*96.45*
PA23	98.54	*99.41*	82.59	*93.63*
PE23	98.44	*99.27*	94.75	*97.41*
PM23	97.53	*98.48*	94.20	*96.44*
SA16	99.72	*99.86*	91.07	*95.57*
SA23	98.35	*99.24*	81.76	*92.18*

Dataset	PR-AUC (%)		ROC-AUC (%)	
	GUIDANCE2	SERES + GUIDANCE2	GUIDANCE2	SERES + GUIDANCE2
IGIA	67.4	*68.49*	91.38	*91.94*
IGIB	80.66	*86.72*	96.47	*97.38*
IGIC2	*74.44*	73.27	*84.63*	82.51
IGID	75.15	*78.38*	96.44	*97.09*
IGIE	94.6	*95.44*	91.84	*93.49*
IGIIA	78.16	*85.09*	94.50	*96.82*
PA23	99.24	*99.53*	91.48	*94.88*
PE23	99.07	*99.34*	96.72	*97.63*
PM23	98.68	*98.85*	96.93	*97.28*
SA16	99.88	*99.91*	96.22	*97.22*
SA23	99.04	*99.33*	89.93	*93.18*

The empirical study made use of benchmark RNA datasets and curated reference alignments from the CRW database [3]. Results are shown for intronic (“IG” prefix) and non-intronic datasets (“P” prefix and “S” prefix, following “primary” and “seed” nomenclature from the CRW database). For each dataset, we report each method’s PR-AUC and ROC-AUC. For each dataset and pairwise method comparison, the best AUC is shown in italics. Methods, performance measures, table layout, and table description are otherwise identical to Table 5

The runtime overhead of SERES + GUIDANCE2 versus GUIDANCE2 was larger on the empirical datasets compared to the simulation study—at most 2.6 h on the largest empirical datasets, which have 100–200 taxa or so (Additional file 1: Figure S1). The runtime difference between the two methods also varied to a greater degree. Unlike the simulation study, GUIDANCE2’s main memory usage was not consistently better than SERES + GUIDANCE2 on the empirical datasets (Additional file 1: Figure S2). Rather, the two methods had comparable memory usage across the empirical datasets, with a maximum difference of 0.06 GiB. Similar runtime and memory usage comparisons were observed for SERES + GUIDANCE1 and GUIDANCE1, with the former having maximum overhead relative to the latter of 4.2 h and 0.07 GiB.

## Discussion

Re-estimation using SERES resampling resulted in comparable or typically improved support estimates for the application in our study. We believe that this performance advantage is due to the ability to generate many distinct replicates while enforcing the neighbor preservation principle. The latter is critical for retaining sequence dependence which is inherent to the application in our study.

On all model conditions, SERES + GUIDANCE1 support estimation resulted in significant improvements in PR-AUC and ROC-AUC compared to GUIDANCE1. A similar outcome was observed when comparing SERES + GUIDANCE2 and GUIDANCE2. The main difference in each comparison is the resampling technique—either SERES or standard bootstrap. Our findings clearly demonstrate the performance advantage of the former over the latter. SERES accounts for intra-sequence dependence due to insertion and deletion processes, while the bootstrap method assumes that sites are independent and identically distributed. Regarding comparisons involving GUIDANCE2 versus GUIDANCE1, a contributing factor may have been the greater AUC of GUIDANCE2 over GUIDANCE1. We used SERES to perform semi-parametric support estimation in conjunction with the parametric support techniques of GUIDANCE1 or GUIDANCE2. The latter method’s relatively greater AUC may be more challenging to improve upon. Finally, the performance of SERES-based support estimation was largely robust to input MSA accuracy as well as algorithmic design choices concerning anchor length and number of anchors. We attribute the latter to the conservative anchors used in the SERES framework, which suffice for the purpose of random walk synchronization and are otherwise not used.

The performance comparisons on empirical benchmarks were consistent with the simulation study. In terms of ANHD and gappiness, the non-intronic datasets in our empirical study were more like the low divergence model conditions in our simulation study, and the intronic datasets were more like the higher divergence model conditions. Across all empirical datasets, SERES-based support estimation consistently yielded comparable or better AUC versus GUIDANCE1 or GUIDANCE2 alone. The SERES-based method’s AUC advantage generally increased as datasets became more divergent and challenging to align—particularly when comparing performance on non-intronic versus intronic datasets. We found that the support estimation methods returned comparable AUC (within a few percentage points) on datasets with 1–2 dozen sequences and low sequence divergence relative to other datasets. In particular, IGIC2 was the only dataset where SERES + GUIDANCE2 did not return an improved AUC relative to GUIDANCE2. IGIC2 was the second-smallest dataset—about an order of magnitude smaller than all other datasets except the IGID dataset—and IGIC2 also had the second-lowest ANHD and lowest gappiness among intronic datasets. IGID was the smallest dataset, but had higher ANHD and gappiness compared to the IGIC2 dataset. Compared to the other empirical datasets, SERES + GUIDANCE2 returned a small AUC improvement over GUIDANCE2 on the IGID dataset—at most 3.2%.

On simulated and empirical datasets, greater sequence divergence generally resulted in a degradation of method performance. However, the SERES-based method’s performance tended to degrade more slowly than the corresponding non-SERES-based method as sequence divergence increased, and the greatest performance advantage was seen on the most divergent model conditions and empirical datasets.

Augmenting GUIDANCE1 and GUIDANCE2 with SERES-based resampling and re-estimation generally increased computational runtime in our study. The added overhead amounted to a few minutes on the 10-taxon and 50-taxon simulated datasets, and grew to a few hours on larger empirical datasets with around 100–200 taxa. In the simulation study, the SERES-based methods also required more main memory than GUIDANCE1 and GUIDANCE2. The gap between the SERES-based methods and standalone GUIDANCE1/GUIDANCE2 appeared to narrow on the larger empirical datasets with a few hundred taxa. Compared to standalone GUIDANCE1/GUIDANCE2, the SERES-based methods perform an additional MSA re-estimation step which occurs after SERES random walk resampling. This difference is likely the primary explanation for the observed computational overhead. We note that the resampling and re-estimation pipelines in our study do not explicitly address scalability, but existing scalability-enhancing techniques can be readily applied to help mitigate added overhead. One option would be to utilize parallelism in the form of pleasantly parallel computation or more sophisticated alternatives (e.g., coordinated and distributed re-estimations that are conditionally independent given a common model instance, parallelized divide-and-conquer algorithms, etc.).

Finally, we note that non-parametric/semi-parametric resampling techniques are orthogonal to parametric alternatives. Consistent with previous studies [18, 20], we found that combining two different classes of methods yielded better performance than either by itself.

## Conclusions

This study introduced SERES, which consists of new non-parametric and semi-parametric techniques for resampling biomolecular sequence data. Using simulated and empirical data, we explored the use of SERES resampling for support estimation involving a classical problem in computational biology and bioinformatics. We found that SERES-based support estimation yields comparable or typically better performance compared to state-of-the-art approaches.

We conclude with possible directions for future work. First, the SERES algorithm in our study made use of a semi-parametric resampling procedure on unaligned inputs, since anchors were constructed using progressive multiple sequence alignment. While this approach worked well in our experiments, non-parametric alternatives could be substituted (e.g., unsupervised *k*-mer clustering using alignment-free distances [4]) to obtain a purely non-parametric resampling procedure. Second, the unaligned input application focused on nucleotide-nucleotide homologies to enable direct comparison against existing MSA support estimation procedures (i.e., GUIDANCE1 and GUIDANCE2). The SERES framework can be extended in a straightforward manner to estimate support for nucleotide-indel pairs. Third, SERES resampling can be used to perform full MSA inference. One approach would be to analyze homologies that appeared in re-estimated inferences across resampled replicates, without regard to any input alignment. Fourth, in the case where biomolecular sequences evolved under insertion/deletion processes, we consider the distinction between aligned and unaligned inputs to be an unnecessary dichotomy. In theory, the latter subsumes the former. We can apply this insight using a two-phase approach: (1) perform SERES-based re-estimation on unaligned sequences to estimate support for aligned homologies (from either an input MSA or the de novo procedure proposed above), and (2) perform support-weighted SERES walks on the annotated MSA from the previous stage to obtain support estimates on downstream inference. Alternatively, we can simultaneously address both problems using co-estimation. Fifth, MSA estimation and MSA support estimation are computationally challenging problems. Applications of the SERES framework to large-scale datasets requires further investigation as part of future algorithmic design studies. Finally, we envision many other SERES applications. Examples in computational biology and bioinformatics include protein structure prediction, detecting genomic patterns of natural selection, and read mapping and assembly. Non-parametric resampling for support estimation is widely used throughout science and engineering, and SERES resampling may similarly prove useful in research areas outside of computational biology and bioinformatics.

## Supplementary information

**Additional file 1.**

## Data Availability

Open-source software and open data can be found at https://gitlab.msu.edu/liulab/SERES-Scripts-Data.
