# Supplementary material for: Non-parametric and semi-parametric support estimation using SEquential RESampling random walks on biomolecular sequences
Source: Algorithms Mol Biol. 2020 Apr 16;15:7. doi: 10.1186/s13015-020-00167-0 (PMC7164268; doi:10.1186/s13015-020-00167-0)
Supplement: Supplementary file 1 — Additional file 1. [file 13015_2020_167_MOESM1_ESM.pdf]

RESEARCH

Additional file 1: non-parametric and semi-parametric support estimation using SEquential RESampling random walks on biomolecular sequences

Wei Wang[1], Jack Smith[1], Hussein A Hejase[2] and Kevin J Liu[1*]

[*]Correspondence: kjl@msu.edu
[1]Department of Computer Science and Engineering, Michigan State University, 48824 East Lansing, MI, USA
Full list of author information is available at the end of the article

Additional methods

SERES walks on aligned sequences

The pseudocode for a non-parametric SERES walk on a fixed MSA A is shown in Algorithm 1.

Algorithm 1 SERES walk on aligned sequences

```
procedure SERESWalkOnAlignedSequences(A, γ, numReplicates)
        ▷ Input: MSA A, walk reversal probability γ, number of SERES replicates numReplicates
        ▷ Output: list of SERES replicates
    replicates = <>
    for i = 1 to numReplicates do
        direction = (rand() > 0.5) ? +1 : −1    ▷ Uniformly at random (UAR) choose direction (right vs. left)
        i = ⌊ length(A) * rand() ⌋ + 1    ▷ UAR draw from [1, length(A)]
                ▷ rand() returns floating point number sampled UAR from [0, 1)
        replicate = <>
        while length(replicate) < length(A) do
            replicate .= A_i    ▷ read A_i, which is the ith character in alignment A
                    ▷ Alignment characters A_i are one-indexed
            i+= direction
            if (i ≤ 0) or (i > length(A)) or (rand() < γ) then
                    ▷ Reflection of random walk
                direction *= -1
                if (i ≤ 0) or (i > length(A)) then
                    i+= direction * 2    ▷ Always reflect at start/end of alignment A
        replicates .= replicate
    return(replicates)
```

SERES walks on unaligned sequences

The pseudocode for SERES resampling of a set of unaligned sequences S is shown in Algorithms 2 through 4.

Algorithm 2 SERES resampling of unaligned sequences

```
procedure SERESWalkOnUnalignedSequences(S, γ, numReplicates)
    ▷ Input: set of unaligned sequences S, walk reversal probability γ, number of SERES replicates numReplicates
    ▷ Output: list of SERES replicates
    replicates = <>
    barriers = <>
    A_init = ObtainGuideAlignments(S)                          ▷ See Algorithm 3
    Ψ = GetAnchorsFromGuideAlignments(S, A_init)               ▷ See Algorithm 3
    AddTrivialBarriers(barriers)
    for each (a⃗, b⃗) Ψ do
        barriers .= a⃗ . b⃗
    for i = 1 to numReplicates do
        replicates .= SERESWalkOnUnalignedSequences(S, γ, i, barriers)
    return(replicates)

static variable maxReplicateLengthFactor    ▷ Maximum replicate length is factor of longest unaligned sequence length
procedure SERESWalkOnUnalignedSequences(S, γ, replicateNum, barriers)
    direction = (rand() > 0.5) ? +1 : −1                       ▷ UAR choose direction (left vs. right)
    i = ⌊ length(barriers) * rand() ⌋ + 1
    replicate = <>
    while maxLength(replicate) < maxLength(S) * maxReplicateLengthFactor do    ▷ maxLength(S) is length of longest unaligned sequence in S
        if ((i == 1) and (direction == −1)) or ((i == length(barriers)) and (direction == +1)) then    ▷ reflect at first or last barrier
            direction *= −1
        AsynchronousReadBetweenAdjacentBarriers(S, barriers, i, direction, replicate)    ▷ read result passed by reference to mutable object replicate
        i += direction
        if rand() < γ then                                     ▷ change walk direction with probability γ
            direction *= −1
    return(replicate)

procedure AsynchronousReadBetweenAdjacentBarriers(S, barriers, i, direction, replicate)
    j = i + direction
    for z = i to n do
        replicate[z] .= (direction > 0) ? substr(S[z], barriers[i], barriers[j]) : reverse(substr(S[z], barriers[j] + 1, barriers[i] + 1)
            ▷ substr(x, i, j) returns substring in index interval [i, j) if i < j or empty string if i ≥ j
    return                                                     ▷ read result passed by reference to mutable object replicate
```

Algorithm 3 Obtain anchors

static variable M ▷ MSA methods $M =< M1, M2, \ldots >$
procedure ObtainGuideAlignments(S)
  alignments $= <>$
  for each (m) M **do**
    alignments $.= m(S)$
  return(alignments)

procedure GetAnchorsFromGuideAlignments(S, A_{init})
  $\alpha = <>$
  $\beta = <>$
  canonicalAlignment $= A_{\text{init}}[1]$ ▷ anchors are indexed based on a fixed alignment in A_{init} (WLOG chosen to be the first alignment in A_{init})
  C_{strict} = GetStrictConsensusColumns(A_{init}) ▷ GetStrictConsensusColumns() returns column indices into first alignment in canonicalAlignment
  $\vec{\alpha}_{\text{strict}}$ = MergeAdjacentColumns(A_{init}, C_{strict}) ▷ merges adjacent columns ▷ returns array of ordered pairs $(\vec{x}, \vec{y})$ where start indices $\vec{x}$ and end indices $\vec{y}$ are indexed based on canonicalAlignment
  SortAnchors($\vec{\alpha}_{\text{strict}}$, canonicalAlignment)
  for $z = 1$ to length($\vec{\alpha}_{\text{strict}}$) **do**
    for $i = 1$ to n **do**
      $(\vec{x}, \vec{y}) = \vec{\alpha}_{\text{strict}}[z]$
      if substr(canonicalAlignment[i], $\vec{x}[i]$, $\vec{y}[i]$) contains only indels **then**
        $\alpha[i][z]$ = LookupUnalignedSequenceIndex(GetLastNonIndelIndexInPrefix(canonicalAlignment[i], $x[i]$))
        $\beta[i][z] = \alpha[i][z]$
      else
        $\alpha[i][z]$ = LookupUnalignedSequenceIndex(GetFirstNonIndelIndexInRange(canonicalAlignment[i], $x[i]$, $y[i]+1$))
        $\beta[i][z]$ = LookupUnalignedSequenceIndex(GetLastNonIndelIndexInRange(canonicalAlignment[i], $x[i]$, $y[i]+1$))
  return(α, β)

procedure SortAnchors($\vec{\alpha}$, canonicalAlignment)
  ▷ $\vec{\alpha}$ is an array of ordered pairs $(\vec{x}, \vec{y})$ where start indices $\vec{x}$ and end indices $\vec{y}$ are indexed based on canonicalAlignment
  sort (ComputeModifiedHammingDistance(u, canonicalAlignment) $<=>$ ComputeModifiedHammingDistance(v, canonicalAlignment)) $\vec{\alpha}$
  ▷ perl sort syntax
  ▷ See Algorithm 4

Algorithm 4 Modified Hamming distance calculation

procedure ComputeModifiedHammingDistance(u, A)
  dist $= 0$
  $(\vec{x}, \vec{y}) = u$
  for $i = 1$ to n **do**
    for $j = i+1$ to n **do**
      dist += ComputeModifiedHammingDistancePair(substr($A[i]$,$\vec{x}[i]$,$\vec{y}[i]$), substr($A[j]$,$\vec{x}[j]$,$\vec{y}[j]$))
  return(dist / $\binom{n}{2}$)

procedure ComputeModifiedHammingDistancePair(x, y)
  alignedLength = length(x) ▷ aligned sequences x and y have same length
  matches $= 0$
  for $i = 1$ to alignedLength **do**
    if ($x[i]$!= INDEL) **and** ($y[i]$!= INDEL) **and** (x[i] == y[i]) **then** ▷ homologies involving indels are penalized as mismatch
      matches++
  return(matches / alignedLength)

Additional figures

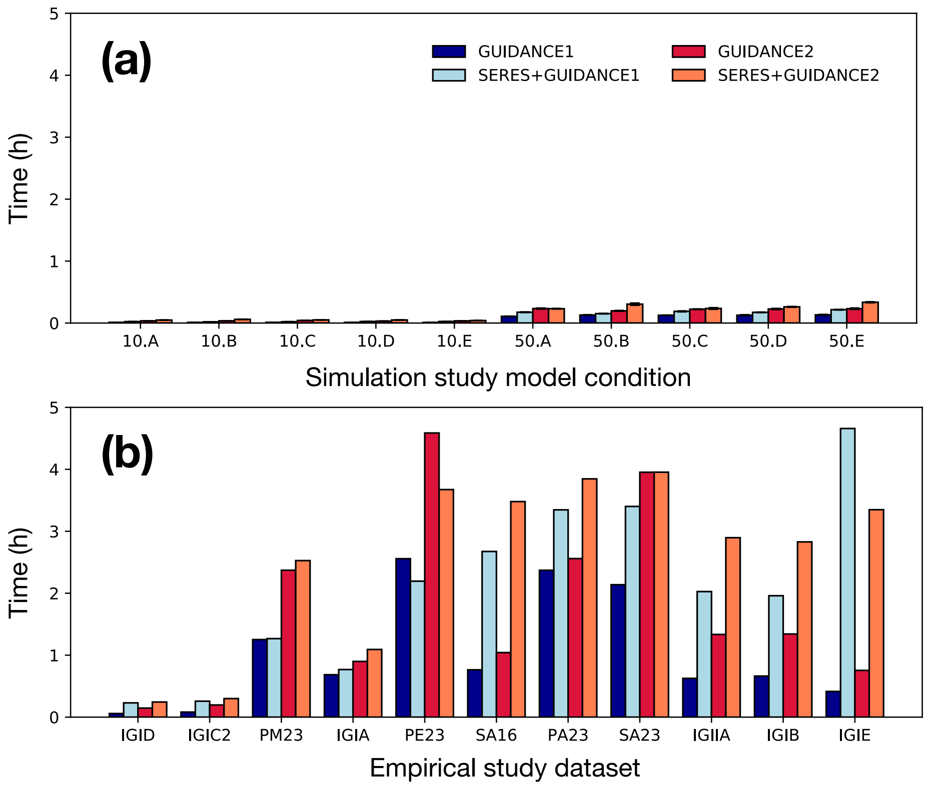

Figure S1 Runtime comparison of methods under study. (a) For each method, average runtime (h) across all replicates in each simulation study model condition is reported ($n = 20$); standard error bars are also shown. The 10-taxon model conditions 10.A through 10.E are shown in order from left to right, followed by the 50-taxon model conditions similarly. (b) Method runtimes are shown for each empirical study dataset. Datasets are arranged from left to right in order of increasing dataset size as measured by number of taxa.

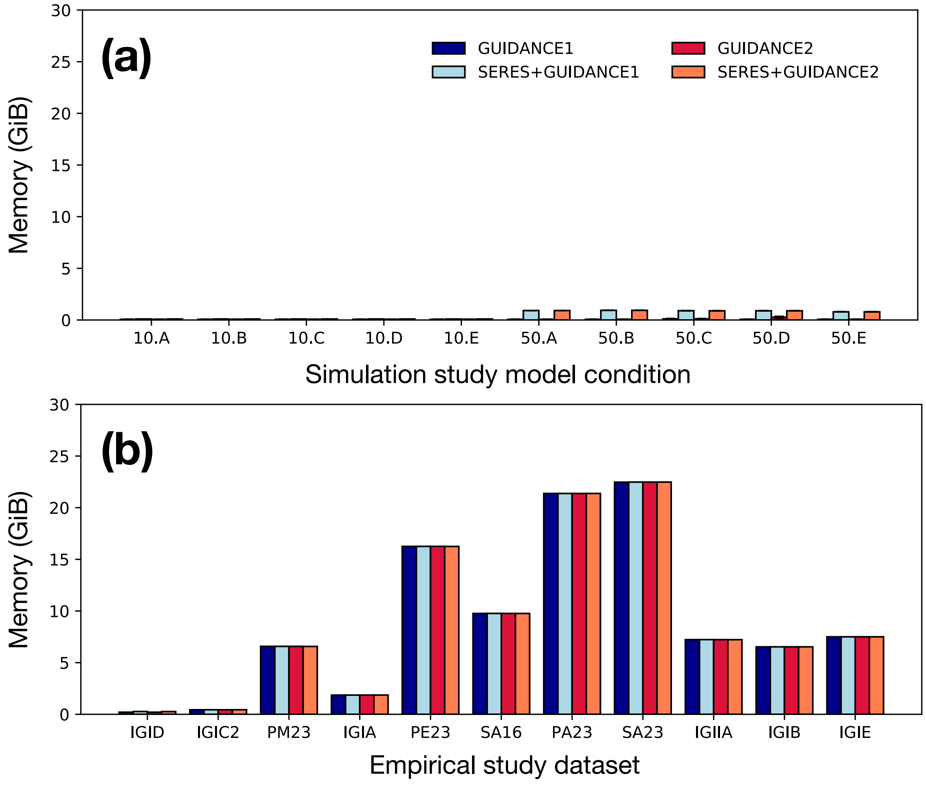

Figure S2 Memory usage comparison of methods under study. Memory usage is shown in GiB. Otherwise, figure layout and description are identical to Supplementary Figure S1.
